# Atrial electrophysiology in hypertrophic cardiomyopathy: current insights and knowledge gaps

**DOI:** 10.1007/s10840-026-02303-z

**Published:** 2026-04-06

**Authors:** Magdalena Klis, Steven E. Williams, Mark O’Neill, Zohya Khalique

**Affiliations:** 1 School of Biomedical Engineering and Imaging Sciences, King’s College London, Westminster Bridge Road, London, SE1 7EH UK; 2 Centre for Cardiovascular Science, University of Edinburgh, Edinburgh, UK; 3 Guy’s and St. Thomas’ NHS Foundation Trust, London, UK

**Keywords:** Hypertrophic cardiomyopathy, Atrial fibrillation, Atrial remodelling, Atrial electrophysiology, Atrial cardiomyopathy, Catheter ablation

## Abstract

Hypertrophic cardiomyopathy is the most common inherited cardiac condition, associated with increased risks of heart failure, arrhythmias, and sudden cardiac death. Once considered a disease of the ventricular myocardium, growing evidence points towards atrial involvement, with structural and electrical atrial remodelling driven by a complex interplay of genetic, electrophysiological and hemodynamic factors. Despite advances in the understanding of this atrial substrate, management of atrial arrhythmias in hypertrophic cardiomyopathy remains challenging. Pharmacological therapies are limited by safety concerns, and catheter ablation shows lower efficacy in patients with hypertrophic cardiomyopathy compared to patients with structurally normal hearts. This review synthesizes current knowledge of disease related changes underlying atrial arrhythmias in hypertrophic cardiomyopathy and outlines diagnostic and therapeutic strategies. Particular emphasis is given to interventional treatment approaches, including emerging technologies and their evolving role in patients with hypertrophic cardiomyopathy.

## Introduction

Hypertrophic cardiomyopathy (HCM) affects 1 in 500 individuals [1], and is characterised by left ventricular (LV) hypertrophy not explained by loading conditions. While hypertrophy is the hallmark feature of HCM, additional histopathological changes such as myocyte disarray, small-vessel disease, and interstitial fibrosis are commonly observed [1]. The disease is predominantly inherited in an autosomal dominant pattern through mutations in genes encoding sarcomeric proteins [2]. The clinical presentation and prognosis are heterogenous, but the condition is associated with increased risk of heart failure, arrhythmias and sudden cardiac death (SCD). Previously considered a disease of the ventricular myocardium, there is increasing evidence of primary atrial involvement in HCM. Diagnostic advances are providing new insights into the arrhythmic atrial substrate in HCM, and the contribution of atrial electrophysiology to arrhythmia development is increasingly recognised.

## Clinical relevance

Atrial fibrillation (AF) affects approximately 20% of patients with HCM, making it the most common sustained arrhythmia in this group of patients [3, 4]. Atrial fibrillation is 4–6 times more prevalent in HCM than in the general population [5] and is associated with increased morbidity and mortality [4, 6]. Patients with AF and HCM face elevated risk of all-cause mortality, SCD, stroke- and heart failure-related death compared to those without AF, despite improvement in overall survival in HCM with contemporary pharmacological and interventional therapies [3, 7]. Newly diagnosed AF is associated with a three-fold increase in the risk of SCD and a doubling of the risk of non-sudden cardiac death, with stroke being the leading cause of non-sudden cardiac death. SCD risk is greater among men, patients with coronary artery disease or patients with a history of heart failure, while non-sudden cardiac death is more common in older patients, women, and those with hyperlipidaemia or prior stroke [8]. AF in HCM often presents early, with nearly one-third of first episodes occurring in patients before the age of 50, although it remains rare in individuals under 30 [9]. Progression of the arrhythmia is typically rapid, with an average of just 7 ± 6 paroxysmal episodes before permanent atrial fibrillation develops [3]. Data comparing morbidity and mortality in patients with paroxysmal, persistent and permanent AF are inconsistent [3, 10], suggesting that adverse outcomes cannot be attributed solely to the loss of atrial contraction. If loss of atrial systole was the only driver, patients with paroxysmal AF, who retain intermittent atrial contractile function, would be expected to have consistently better outcomes.

Despite advances in HCM management, AF remains a marker of disease progression and a therapeutic challenge with electrical and mechanical atrial remodelling in HCM likely creating a vulnerable substrate for AF [11]. Conventional treatment strategies offer limited efficacy with long-term freedom from arrhythmia after a single AF catheter ablation being reported at just 45% [12]. The limited efficacy of such treatment strategies may be accounted for by these mechanical and electrical alterations and underscore the need for deeper understanding and dedicated diagnostic and therapeutic approaches.

## Cellular, structural and functional evidence for HCM-associated atrial cardiomyopathy

### Cellular electrophysiological alterations in HCM

Once considered a purely structural heart disease, HCM is now recognised to include important electrophysiological abnormalities of many transmembrane and intracellular ionic currents [13, 14].

Patch-clamp studies show prolonged action potential duration (APD) in HCM myocytes, driven by increased I_NaL_ and L-type calcium current (I_CaL_), reduced repolarising potassium currents (I_to_, I_Kslow1_, I_K1_, I_Ks_ and I_Kr_) and slower calcium transient kinetics. The increase in sodium and calcium currents appears early in disease progression, and may reflect a direct effect of sarcomeric mutations [15]. Elevated intracellular sodium disrupts the Na⁺-Ca²⁺ exchanger (NCX), reducing diastolic Ca²⁺ extrusion and promotes systolic Ca²⁺ influx, particularly at higher pacing frequencies [16]. Reduced sarco/endoplasmic reticulum Ca²⁺-ATPase (SERCA, the pump responsible for calcium reuptake into the sarcoplasmic reticulum) activity, impaired NCX function, and disorganised T-tubules further promote calcium overload. Increased myofilament calcium binding contributes to elevated end-diastolic calcium concentration and potentiates calcium release from the sarcoplasmic reticulum. This results in increased likelihood of spontaneous Ca²⁺ release via ryanodine receptors [17], increasing risk of delayed afterdepolarisations (DADs).

Prolonged APD also promotes calcium channel reactivation during repolarisation, resulting in early afterdepolarisations (EADs). EADs typically occur at low stimulation frequencies while DADs are more common at higher rates [18]. Both contribute to triggered activity, particularly under β-adrenergic stimulation when I_CaL_ inactivation is further delayed [19].

Animal models provide further evidence. Chronically increased Ca²⁺ sensitivity in troponin T mutation mice resulted in an increased frequency of EADs at high pacing rates and triggered beats after pauses [20]. Myosin heavy chain mutation myocytes exhibit frequent triggered activity, even at low stimulation frequencies, amplified by isoproterenol. They also showed prolonged APD, reduced connexin-43 expression, impaired repolarisation, and incomplete membrane recovery at higher heart rates [21]. Loss of K⁺ currents also contributes to slowed repolarisation and prolonged refractory periods, creating a pro-arrhythmogenic substrate [22]. Animal models of MYBPC3 and MYH7 mutations show QTc prolongation resulting from reduced repolarising K⁺ channels even in the absence of detectable hypertrophy [23]. Regional differences in potassium current remodelling additionally contribute to repolarisation dispersion, increasing proarrhythmic risk [24]. Ion channel changes seen in HCM and their effect on APD are summarised in Table 1. 

**Table 1:**
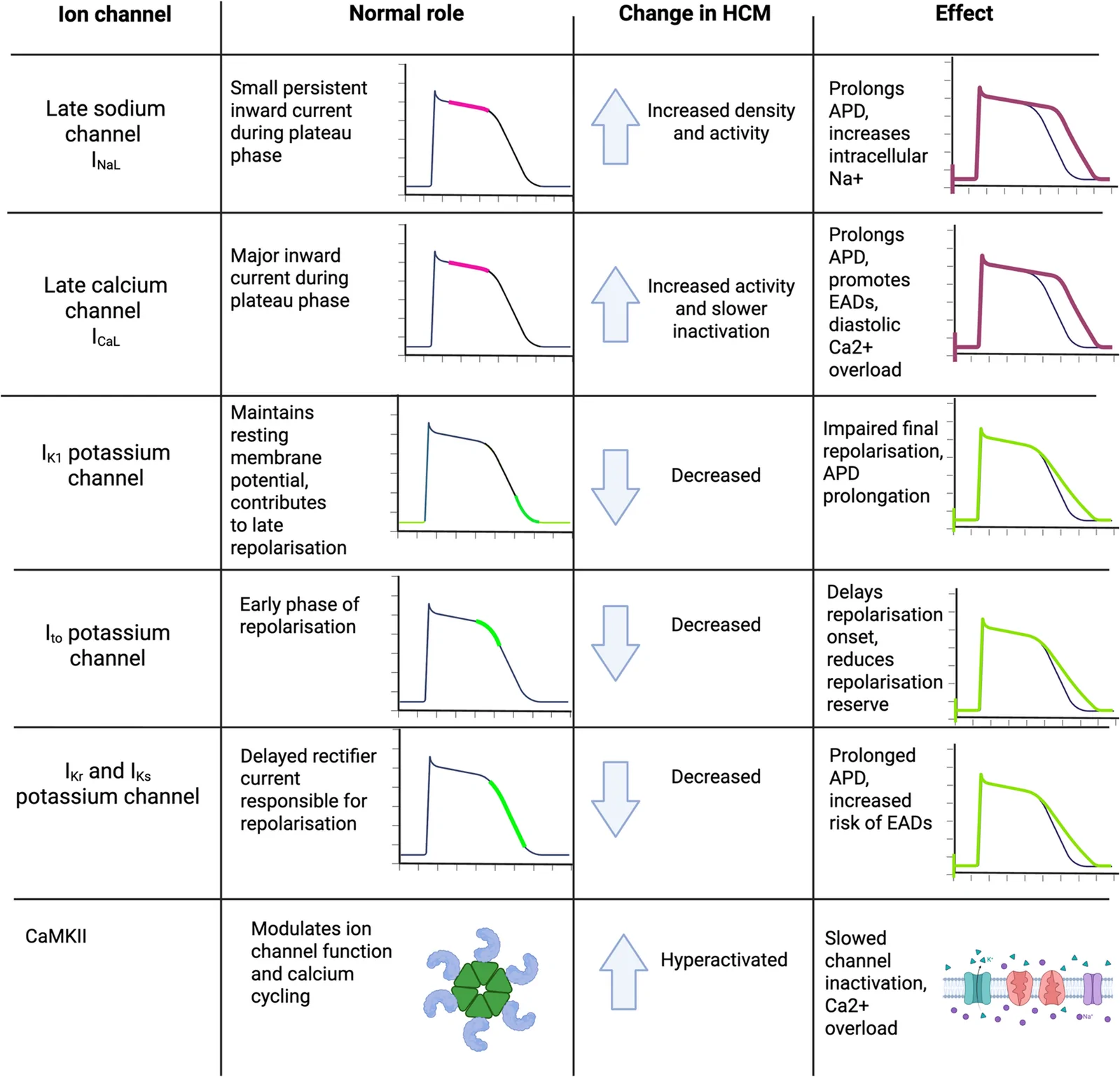
Summary of ion channel changes seen in HCM and their effect on action potential

**Fig. 1:**
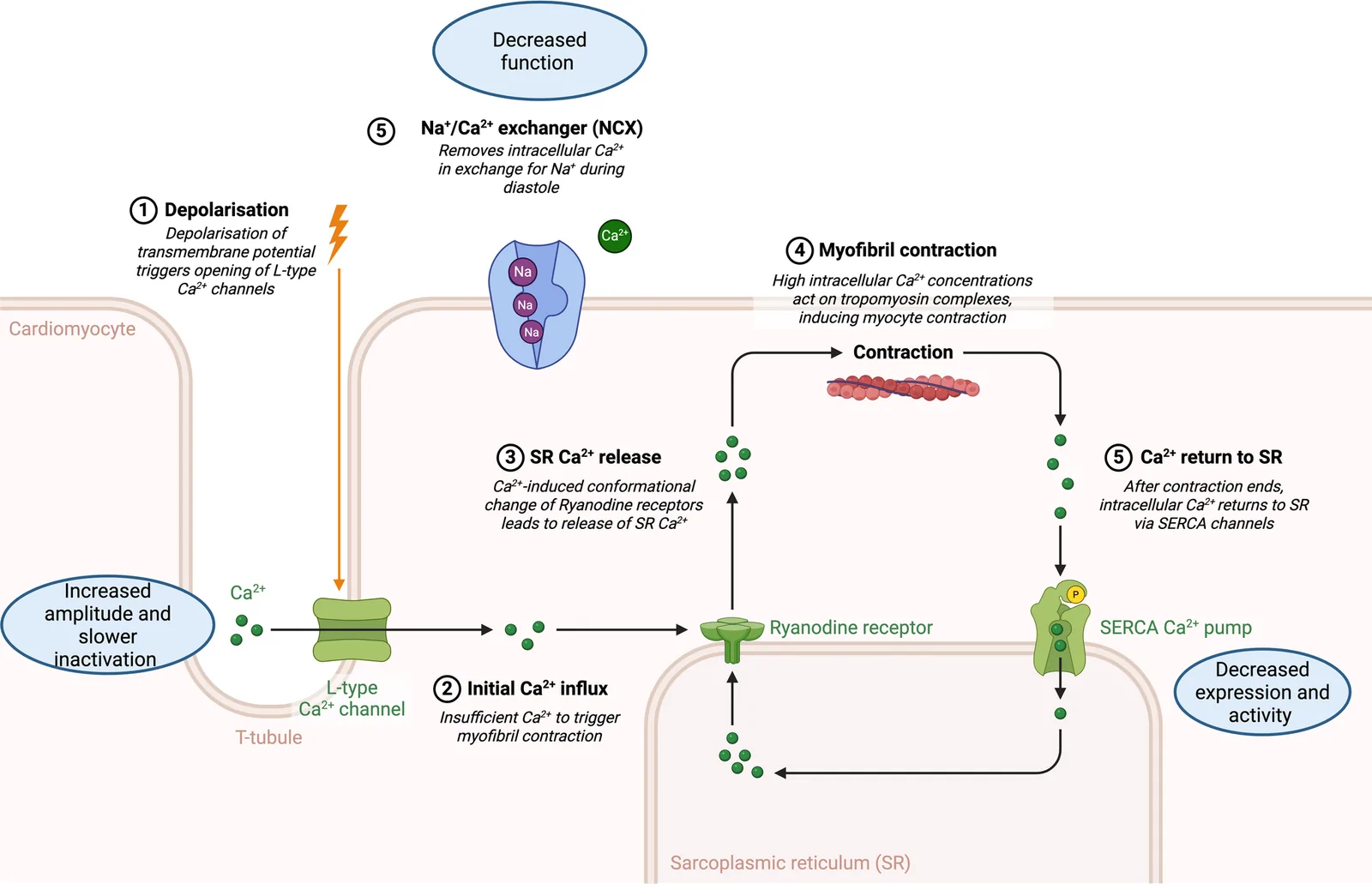
Diagram of intracellular calcium kinetics with changes seen in HCM in blue circles

These changes are linked to activation of calcium/calmodulin-dependent protein kinase II (CaMKII), which regulates calcium handling, ion channel function, ryanodine receptors and excitation–contraction coupling [25, 26]. In sarcomere-mutation HCM, raised CaMKII levels and activity lead to altered channel gating, elevated intracellular Na + and Ca^2+^ levels and impaired calcium reuptake. In parallel, the expression of SERCA is reduced in cardiac tissue from both gene-positive and gene-negative patients with HCM [27]. Despite robust animal data, human studies are less consistent, with many cell models and surgical tissues failing to show APD prolongation, suggesting species differences or phenotypic variability [22]. Figure 1 is a schematic representation of changes in calcium kinetics in HCM.

Autonomic dysfunction also affects electrophysiological properties in HCM. β-stimulation paradoxically prolongs APD by reducing I_Ks_, increasing I_CaL_ and slowing its inactivation [28]. Abnormal cardiac norepinephrine reuptake and myocardial β-adrenergic receptor downregulation increase local catecholamine levels [29], enhancing automaticity and promoting EADs and DADs [30].

Increased calcium sensitivity also causes localised energy deficits during isoproterenol infusion or rapid pacing, disrupting intercellular coupling and slowing transverse conduction, heightening conduction anisotropy and the arrhythmic vulnerability [14].

### Atrial structural changes in HCM

Emerging evidence suggests that histopathological changes characteristic of HCM, such as myocyte disarray, interstitial fibrosis, and microvascular dysfunction, extend beyond the ventricles to involve the atria. Atrial remodelling appears distinct from that seen in other conditions - LA function is reduced compared to both hypertensive LV hypertrophy and healthy controls [31], and more advanced than in phenocopies like Fabry disease [32]. On a cellular level myocyte hypertrophy and disarray have been described in both atria of patients with HCM [33].

On a cellular level a pro-fibrotic and inflammatory state appears to drive disease progression even before hypertrophy is detectable. Inflammation and fibrosis have been shown in murine models [34], and sarcomeric mutation carriers without hypertrophy exhibit elevated biomarkers of collagen synthesis and early TGF-β-mediated profibrotic signalling [35]. An inflammatory response within the myocardium of patients with HCM has also been confirmed on endomyocardial biopsy samples [36]. In mice this proliferative and profibrotic signalling can be suppressed by TGF-β-neutralising antibodies and angiotensin II receptor antagonists [37].

These structural changes create a substrate for arrhythmias by disrupting conduction. Activated myofibroblasts couple with myocytes via gap junctions, depolarising resting potential and reducing Na^+^ channel availability [38]. In patients without HCM, fibrosis encases myocytes in collagen, slowing transverse but accelerating longitudinal conduction [39, 40]. In vitro HCM models, using stem cell-derived cardiomyocytes with MYH7 mutation, show altered gap junction distribution due to decreased connexin-43 expression which significantly reduces transverse conduction velocity [41] especially at short cycle lengths, enabling small circuit re-entry and leading to AF [42].

The phenotype of atrial disease evolves with age and loading conditions [43]. Younger patients with HCM exhibit more pronounced myocyte hypertrophy and disarray, while older patients show more extensive fibrosis and endocardial thickening. This may explain the clinical progression from ventricular arrhythmias in the young to AF and heart failure in later life [44, 45]. Furthermore, increased epicardial fat volume in patients with HCM correlates with presence of AF, suggesting potential paracrine or autocrine effect [46].

Despite histological evidence of myocyte hypertrophy and surgical observations of thickened atrial tissue during Maze procedures [47], a study assessing left atrium (LA) wall thickness using CT found no significant increase in LA wall thickness compared to controls, and even decreased posterior wall thickness [48], possibly reflecting atrial stretch secondary to elevated pressures or fibrotic remodelling.

Technological advances in cardiac imaging have significantly improved the characterisation of atrial remodelling in HCM. Several factors predict major AF outcomes in HCM, including age, higher BMI (particularly in younger patients), increased LA volume and reduced atrial contractile function on cardiac MRI (CMR) (especially in middle-aged and older patients), and the presence of moderate or severe mitral regurgitation. A CMR study assessing LGE in the left atrium identified fibrosis in all the HCM patients [49], with a predilection for the posterior wall, especially near the left inferior pulmonary vein. A greater extent of LA LGE is associated with impaired LA compliance and reduced reservoir strain, providing a structural basis for arrhythmogenesis [49]. Incorporating CMR assessment of atrial volume and function may enhance AF related risk stratification [50]. Meta-analyses have shown that in all patients with AF every 10% increase in LA fibrosis quantified by cardiac MRI results in a 1.5-fold rise in AF recurrence following ablation, highlighting its strong prognostic value [51]. In patients with HCM, LV LGE is also associated with increased risk of AF [52]. Among patients with normal LA volume index (< 34 mL/m²), increased AF prevalence has been reported when ventricular LGE exceeded 15% [53], likely reflective of higher arrhythmia prevalence in more advanced stages of the disease [52].

LGE on CMR primarily detects replacement fibrosis and correlates only weakly with interstitial fibrosis [54]. Histology, on the other hand, reveals both replacement and interstitial fibrosis, the latter more closely correlating with AF risk [45]. Electroanatomic voltage mapping, which reflects functional impact of atrial myopathy, demonstrates variable correlation with LGE-detected fibrosis in patients with AF and structurally normal hearts [55]. This reflects the limitations of imaging in detecting diffuse microscopic changes contributing to arrhythmogenesis.

The breadth of pre-clinical and clinical data supports a wide spectrum of structural atrial changes amongst patients with HCM. It remains unclear which directly affect atrial arrhythmia susceptibility, and which reflect progression of HCM without increasing arrhythmia risk.

### Significance of raised left atrial pressure

Elevated LA pressure is a well-recognised risk factor for the development of AF in both patients with and without HCM. However, there remain unique haemodynamic contributors and consequences of HCM to LA pressure and subsequent atrial remodelling.

In HCM, increased LV wall thickness impairs diastolic filling leading to elevated LA pressure. The resulting atrial stretch and remodelling may shorten the effective atrial refractory period and increase dispersion of depolarisation, both potentially creating a substrate for AF. However, impaired LA function has also been observed in carriers of sarcomeric gene mutations, independent of LV filling pressure and LA pressure, suggesting a genetic contribution to atrial dysfunction and arrhythmogenesis may exist in HCM [56].

Nevertheless, patients with obstructive physiology are at particularly high risk of developing AF, emphasising the role of chronic pressure overload. Surgical reduction of the outflow gradient through septal myectomy appears to attenuate this risk: post-myectomy patients have a similar incidence of AF to nonobstructive HCM, underscoring the value of early intervention. Nonetheless, atrial remodelling may persist or progress post-operatively, with up to 21% of patients developing de novo AF even after septal reduction surgery [57].

These findings reinforce the multifactorial basis of AF in HCM, where hemodynamic stress may be just one contributor to arrhythmia risk, alongside genetic predisposition and intrinsic atrial myopathy.

### Genotype-phenotype correlation

While LA dilatation is strongly associated with AF in HCM [58], genetic factors also play an important role. Certain sarcomeric gene variants – particularly in *MYH7* and *TNNT2* – have been associated with a higher intrinsic risk of AF, independent of LA size. Cohort studies show that *MYH7* pathogenic variants associate with the highest incidence of AF, even after adjusting for age, sex, LA size, maximal wall thickness and peak pressure gradient compared with MYBPC3 or thin filament mutations [59].

Various mutations appear to have distinct effects – murine models of troponin I mutation exhibit increased conduction heterogeneity, reduced connexin-43 expression and structural changes [34], while troponin T mutation (TNNT2) show action potential shortening at 70% repolarisation, with decreased I_K1_ current [60]. Similarly, certain hotspot mutations in TNNT2 are associated with higher atrial arrhythmia burden than sporadic mutations, related to increased myofilament calcium sensitivity, prolonged contraction-relaxation cycles, and a high rate of spontaneous activity. Patients with troponin T mutations also exhibit increased risk of SCD despite minimal hypertrophy and fibrosis [61, 62]. Alpha-actinin-2 mutations often exhibit atrial electrical abnormalities independent of structural changes, as demonstrated in mice models [63].

Patients with MYH7 or MYBPC3 mutations undergoing AF ablation were also found to have more extensive LA low-voltage areas, despite similar LA volumes and pressures as gene-negative counterparts [64]. These observations supports the notion that specific mutations affect atrial arrhythmogenicity beyond haemodynamic effects the disease has on the atria, with important implications for risk stratification [65].

Beyond sarcomeric mutations, polymorphism in the aldosterone synthase gene has been independently linked to AF, likely through aldosterone-induced atrial fibrosis and myocyte apoptosis [66]. Furthermore, polymorphisms in potassium channel genes have also been reported in HCM patients with AF [11]. Although diastolic dysfunction and raised atrial pressure with resulting LA dilatation appear to be central drivers of AF in HCM, polygenic risk scores have shown predictive utility, highlighting the contribution of genetic factors [67].

Together, these insights suggest that integration of genetic data into future studies of AF risk may allow improved prediction of atrial arrhythmias in HCM.

## Impact on clinical atrial electrophysiology

Structural and electrical remodelling in HCM affects electrophysiological properties of the atria [68], impairing electrical coupling between myocytes and decreasing local conduction velocity. This creates the conditions necessary for re-entry and sustained atrial arrhythmias. Although direct in vivo human data in patients with HCM have historically been limited, recent studies using electroanatomic mapping have begun to define the specific characteristic of atrial myopathy in HCM.

In a cohort of 28 patients with HCM and AF, electroanatomic mapping during ablation revealed larger low-voltage areas in the left atrium compared to control patients. These low voltage areas were independently associated with atrial arrhythmia recurrence in patients with HCM [69]. In a separate study of 20 patients with HCM undergoing AF ablation, slow conduction regions were identified in the posterior wall even in the presence of normal bipolar voltage, suggesting that altered conduction may be present in the absence of overt fibrosis or low voltage areas [70]. In another study, pre-ablation electroanatomic mapping in 10 patients with HCM demonstrated significantly lower bipolar voltage, larger low-voltage areas and more heterogeneous conduction relative to matched controls with AF and structurally normal hearts, highlighting pronounced conduction abnormalities likely contributing to AF susceptibility [71]. Further evidence for electrical remodelling comes from epicardial mapping in 15 patients with HCM undergoing myectomy for LV outflow tract obstruction. Despite no prior history of AF, areas of slow conduction and low voltage were present in both atria, supporting the presence of a co-existent atrial myopathy [68].

Patient specific computational models based on atrial MRI in three patients with HCM provided further evidence, showing a significantly higher AF initiation rate compared to patients with AF and structurally normal hearts and initiated arrhythmia was more complex as evidenced by a significantly greater number of waves per AF episode [72].

**Fig. 2:**
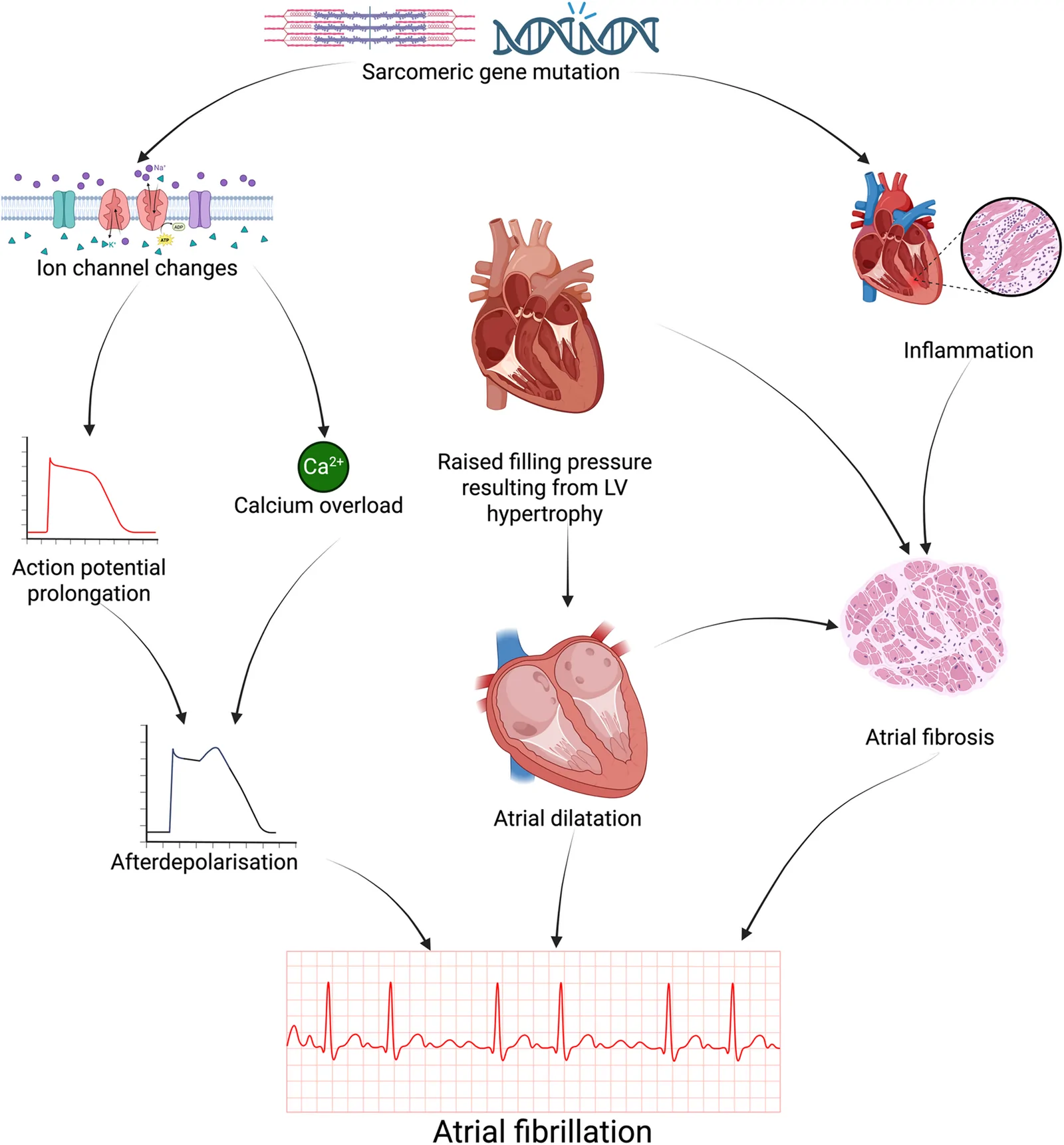
Schematic representation of changes in patients with hypertrophic cardiomyopathy leading to higher prevalence of atrial fibrillation

These findings highlight that atrial electrical dysfunction in HCM stems from both structural and molecular remodelling, which can precede detectable fibrosis and contribute to AF vulnerability. Despite these insights, a significant gap remains in linking specific electrophysiological changes to the underlying mechanisms and long-term outcomes of arrhythmias. Future research integrating high-density mapping with genetic profiling and advanced imaging will be crucial to understand these complex interactions and develop more effective, substrate-specific ablation and treatment strategies for atrial arrhythmias in patients with HCM. Figure 2 is a schematic representation of changes seen in HCM contributing to higher prevalence of AF.

## Therapeutic consequences

### Thromboprophylaxis

Given the high thromboembolic risk in HCM, possibly driven by the presence of atrial myopathy, anticoagulation is recommended for any clinical AF irrespective of CHA₂DS₂-VASc score. Thromboprophylaxis is also advised for subclinical AF lasting more than 24 h, while shorter episodes (5 to 24 h), may require treatment based on individual risk profiles [73].

Interestingly, in patients with implantable cardiac devices and no prior AF history, stroke rates are similar in those with de novo AF and those in stable sinus rhythm. Severe LA dilatation remains a strong predictor of stroke, regardless of the rhythm [74], highlighting the need for improved risk stratification in this group.

Direct oral anticoagulants (DOACs) are increasingly preferred due to their effectiveness and ease of administration. A meta-analysis of observational studies confirmed that DOACs are both safe and effective in HCM patients when compared to warfarin [75].

Despite high rates of anticoagulation, patients with HCM and AF are more likely to have LA thrombus on transoesophageal echocardiography (TOE) compared to non-HCM controls, suggesting the need for low threshold for TOE prior to ablation or cardioversion, even when appropriately anticoagulated [76]. Although a retrospective study found no reduction in stroke rates with TOE-guided cardioversion, the reported 2% stroke rate in both TOE and non-TOE groups remains higher than expected [77].

### Rhythm control

Both the American Heart Association (AHA) and the European Society of Cardiology (ESC) guidelines emphasise that maintaining sinus rhythm is critical in patients with HCM, given the important contribution of atrial contraction to ventricular filling in this population.

The AHA recommends a rhythm control strategy in patients with poorly tolerated AF, utilising cardioversion or antiarrhythmic drugs (AAD) (Class IIa). Catheter ablation is given Class IIa recommendation when drug therapy is ineffective, contraindicated or not the patient’s preference [78].

The ESC states that maintaining sinus rhythm is “highly desirable” and that a rhythm control strategy is preferred, particularly in symptomatic patients. The guidelines further describe catheter ablation as a safe and superior alternative to AADs, which reduces AF-related symptoms, improves quality of life and “can be considered an alternative to AAD therapy in practically any type and context of AF”. Accordingly, ablation is given a class I recommendation in the setting of one failed or not tolerated AAD. A class IIa recommendation is given to ablation as a first line therapy in selected patients without major risk factors for recurrence to improve symptoms, as well as for ablation at an early stage of AF progression, regardless of symptoms [2].

The importance of an early invasive strategy is further reinforced in the EHRA consensus statement, which also supports early intervention to prevent substrate progression and increases success rates [79].

#### Pharmacological treatment options

While amiodarone remains one of the most effective antiarrhythmic drugs used in HCM, its long-term use is limited by side-effects. Discontinuation due to inefficacy is rare but adverse effects limit its use in 19% of patients [80]. Alternative agents like sotalol and dofetilide carry other risks such as bradycardia and QTc prolongation. In a cohort of 72 patients, both dofetilide and sotalol had approximately 30% discontinuation rates, primarily due to inefficacy, and 40% AF recurrence at one year, though heart failure hospitalisation and mortality rates remained low [81]. In another study sotalol was well-tolerated but had a high discontinuation rate due to inefficacy (27.2%) [82].

Disopyramide, a class 1a antiarrhythmic, improves LV obstruction while exerting multichannel blocking properties [83]. Although generally well tolerated, rare but serious adverse events were reported, including anaphylaxis, sustained ventricular tachycardia and QTc prolongation. In the same study, dofetilide was associated with symptomatic bradycardia and syncope [82].

The safety of class Ic agents such as flecainide, propafenone, and quinidine in HCM is not well established, however given their proarrhythmic potential in structural heart disease, these medications are generally avoided in the management of HCM.

Early use of beta-blockers, calcium-channel antagonists, and disopyramide may delay AF onset, though their benefit wanes beyond 2–3 years. Unexpectedly, amiodarone therapy does not appear to prevent the development of AF [5]. While several pharmacological options exist, individualised risk-benefit profile assessment is essential (Table 2).

**Table 2:** Safety and efficacy of medications used for rhythm control in hypertrophic cardiomyopathy

Medication	Class	Mechanism	Efficacy	Limitations/Adverse Effects	Notable Findings
Amiodarone	Class III	Multi-channel blocker	Highly effective in HCM	Long-term use limited by side effects (19% discontinuation) [80]	Did not prevent AF onset in one study [5]
Sotalol	Class III	β-blocker and K⁺ channel blocker	Similar efficacy to dofetilide; ~40% AF recurrence at 1 year	~ 30% discontinuation rate (mostly inefficacy), QTc prolongation, bradycardia	High discontinuation due to inefficacy (27.2%) [82]
Dofetilide	Class III	K⁺ channel blocker	Comparable to sotalol	~ 30% discontinuation; bradycardia, QTc prolongation, syncope	Serious adverse events reported [81]
Disopyramide	Class Ia	Na⁺, K⁺, Ca²⁺ channel blocker; Ryanodine receptor stabiliser; negative inotrope [83]	Effective in obstructive HCM; reduces LVOT gradient and arrhythmias [82]	Risk of QTc prolongation, sustained VT and anaphylaxis	May delay AF onset when used early [5]
Flecainide, Propafenone, Quinidine	Class Ic	Na⁺ channel blockers	Unclear efficacy in HCM	Known proarrhythmic risk; generally avoided in HCM	N/A
Ranolazine	I_NaL_ inhibitor	Reduces intracellular Ca²⁺ overload; Ryanodine receptor stabiliser; mild β-blocking	Promising; reduces ventricular arrhythmic burden in HCM [111]	Limited clinical data	Cellular mechanism supports use [109, 110]
Beta-blockers & Calcium Channel Blockers	Various	Rate control; reduce sympathetic tone or Ca²⁺ influx	May delay AF onset when used early	Benefits wane after 2–3 years	Propensity-matched studies suggest early benefit [5]

*AF* atrial fibrillation, *HCM* hypertrophic cardiomyopathy

**Table 3:** Summary of studies on AF ablation in HCM

Aspect	Key Findings	Clinical Implications
Symptomatic & functional benefit	Reduces AF burden; improves NYHA class and quality of life; lowers incidence of heart failure hospitalisation [85–87]	Meaningful symptomatic and functional improvement
Single-procedure success	39–61% freedom from AF (> 30 s episodes) [88]	Lower than structurally normal hearts
Repeat procedures	Up to 71% success [88]	Multiple ablations often required
PVI vs. PVI + substrate modification	No significant difference in arrhythmia-free survival [92]	Optimal strategy has not been established
Non-PV trigger ablation	Small single-centre study showed 94% arrhythmia-free at 15 months [93]	Limited evidence: findings require confirmation
CFAE ablation	Higher incidence of atrial tachycardia (58.8% vs. 27.7%) [94]	Extensive ablation may promote AT
Cryoballoon vs. radiofrequency	Comparable efficacy; cryoablation associated with shorter procedure time and less fluid volume [95]	Cryoablation may be advantageous in patients prone to fluid overload
VOM ethanol infusion	Addition of VOM ethanol infusion to radiofrequency ablation increases arrhythmia-free survival at 12 months from 65.2% to 84.6% [96]	May improve mitral isthmus block and outcomes
PFA	Higher arrhythmia-free survival at 12 months compared to thermal ablation (67.6% vs. 47.3%); shorter procedure time; lower incidence of post-procedure HF [97]	Promising modality, possibly better lesion homogeneity in fibrotic atria
Surgical ablation	63.2% AF-free at 7 years; Maze IV procedure improved survival vs. myectomy alone [99]	Can be useful in patients with HCM and AF undergoing myectomy [78]
Overall interpretation	Higher recurrence than general AF population, but clinically valuable	Success should be judged by symptom reduction, not just AF elimination

*AF* atrial fibrillation, *AT* atrial tachycardia, *CFAE* complex fractionated atrial electrograms, *HCM* hypertrophic cardiomyopathy, *PFA* pulsed field ablation, *PV* pulmonary vein, *PVI* pulmonary vein isolation, *VOM* vein of Marshall

#### AF ablation

Catheter ablation has not been shown to reduce mortality in HCM, but it lowers AF burden [84], contributing to symptomatic improvement [85], and improved functional status [86]. Long-term data suggest that ablation in this group delays progression of AF, improves NYHA class and quality of life and is associated with lower incidence of heart failure hospitalisation [87].

Meta-analyses show single-procedure success rates (freedom from recurrent atrial arrhythmia lasting longer than 30 s) of 39–61%, improving to 71% with repeat ablations. Recurrence rates remain three-fold higher than in patients with structurally normal hearts [88], often requiring ongoing antiarrhythmic therapy [85, 89, 90]. Outcomes are influenced by AF type, ablation strategy, follow up duration and baseline disease characteristics but a meta-analysis showed that at 5-year follow up, 70% of patients treated with an invasive strategy remained in sinus rhythm [87]. In studies reporting AF duration before ablation, the median was 5.9 years [91], suggesting delayed intervention may be contributing to worse outcomes.

The optimal ablation strategy in HCM patients has also not been established. Current evidence suggest no significant difference in arrhythmia-free survival between patients who underwent pulmonary vein isolation only and pulmonary vein isolation plus substrate modification strategies, regardless of AF type or genotype [92]. However, one study of 43 patients with HCM undergoing repeat ablation showed efficacy of targeting non-pulmonary vein triggers. Despite durable pulmonary veins and posterior wall isolation, late recurrences (mainly atypical atrial flutter) were common with triggers found in the coronary sinus, LA appendage, interatrial septum, and right atrium and were associated with older age, larger LA size, and reduced ejection fraction. While this study reported a 94% arrhythmia-free survival off antiarrhythmic medications at a median 15-month follow-up, these findings, derived from a small, single-centre cohort over a decade ago, are considerably higher than those in the wider literature and should be interpreted with caution [93].

It has been shown that patients with HCM in whom complex fractionated atrial electrograms (CFAE) were identified and ablated were more likely to develop atrial tachycardia (AT) than those with pulmonary vein isolation only (58.8% vs. 27.7%). Electrophysiologically unstable AT during ablation was strongly associated with previous CFAE ablation, while stable AT was usually seen in patients without prior CFAE ablation. While the authors attributed the higher incidence of ATs to a more complex substrate characterised by CFAEs, it is also plausible that higher incidence of AT is a consequence of more extensive ablation rather than CFAEs themselves [94].

Cryoballoon and radiofrequency ablation approaches in HCM show comparable efficacy, with cryoablation offering shorter procedural times and reduced irrigation volumes, particularly important in patients with history of fluid overload, which is common in patients with HCM [95].

The vein of Marshall (VOM) contains autonomic nerves, myocardial fibers and arrhythmogenic foci. It is also located along the mitral isthmus, an obligatory component of perimitral flutter circuit and therefore a common target for ablation. Ablation in this region is technically demanding owing to epicardial fibres in the VOM, heterogeneity in wall thickness and close proximity to the coronary sinus. The addition of VOM ethanol infusion to radiofrequency ablation in patients with HCM led to a modest increase in the rate of achievement of acute mitral isthmus block. However, the benefit of VOM ablation appeared to extend beyond simply achieving mitral isthmus block. After 12 months of follow-up, 84.6% of patients who had additional vein of Marshall ablation remained free from atrial arrhythmias, compared to 65.2% in the radiofrequency ablation only group [96].

More recently pulsed field ablation (PFA) has emerged as a promising modality. In a study of 109 patients with HCM undergoing AF ablation, PFA was compared to thermal ablation. PFA had significantly shorter procedure times and lower rates of post-procedure heart failure. At 12 months, freedom from atrial arrhythmia recurrence was higher with PFA (67.6% vs. 47.3%). Unlike thermal ablation, additional lesions beyond pulmonary veins did not increase arrhythmia recurrence risk. PFA’s effectiveness may be due to more homogeneous lesion formation in fibrotic tissue, which may be particularly relevant in HCM patients. Additionally, shorter procedure times with PFA may help reduce fluid-related complications like heart failure [97].

Surgical ablation may be more effective than catheter ablation in patients with HCM. A meta-analysis of non-randomised studies evaluating surgical ablation during myectomy, primarily utilising the Cox Maze III or IV procedure, reported a 63.2% long-term freedom from AF at seven years [98]. Although this meta-analysis had no true comparator group, the reported freedom from AF recurrence appears higher than in contemporary catheter ablation studies in HCM. The overall risk of bias was moderate, driven mainly by missing data and incomplete outcome reporting. Cox Maze IV procedure performed alongside septal myectomy improved mid-term survival and reduced AF recurrence compared to myectomy alone [99]. Summary of studies on AF ablation in HCM is provided in Table 3.

Despite higher recurrence rates, ablation remains a valuable tool in AF management in HCM, particularly when integrated into a comprehensive rhythm control strategy. It’s important to recognise that the success of AF interventions should not be measured solely by the presence or absence of recurrent episodes, but by the overall reduction in symptom burden - a shift from frequent to less frequent and better-tolerated AF episodes may represent a meaningful and acceptable outcome for some patients [85–87].

### Rate control

Beta-blockers and nondihydropyridine calcium channel blockers are the first line agents for rate control in AF. Digoxin is generally avoided, particularly in obstructive HCM, due to its positive inotropic effect, which may exacerbate LVOT obstruction.

For patients with HCM and inadequate pharmacologic rate control, AV node ablation in conjunction with cardiac implantable device therapy has demonstrated safety and efficacy. In a cohort of 54 patients with HCM it showed over 80% symptomatic improvement. 91.5% showed improvement in EHRA classification, and 86.4% in NYHA classification, with similar benefits seen in both paroxysmal and persistent AF groups. 47.5% of the patients had a device implanted for SCD prevention and 32.3% primarily as part of pace and ablate strategy. Patients undergoing AV node ablation due to inappropriate ICD shocks showed less symptomatic benefit than patients treated due to suboptimal rate control. Presence of cardiac resynchronisation therapy did not affect symptomatic benefit compared to other types of devices. AV node ablation out-performed first-time AF ablation, highlighting the contribution of irregular R-R interval to patients’ symptoms [100]. There are, however, concerns about permanent right ventricular pacing, including potential deterioration of LV function and device-related complications in a pacing-dependent patient. While biventricular pacing alleviated concerns regarding LV function, long-term safety data is lacking, and thus it is absent from the 2020 AHA/ACC guidelines for HCM treatment.

Left bundle branch area pacing (LBBP) is emerging as a more physiological alternative in patients with non-obstructive HCM with bradycardia pacing indications. Importantly, LBBP has been shown not to impair cardiac function and not to increase LVOT gradient or pulmonary artery systolic pressure [101]. However, its feasibility may be limited by septal fibrosis, as evidenced by only 36.4% procedural success rate in one study, where high failure rate was attributed to difficulty penetrating the fibrotic septum or achieving left bundle capture [102].

### Other treatment strategies

Mavacamten, a myosin ATPase inhibitor approved for treatment of obstructive HCM, has been shown to improve symptoms and reduce LVOT obstruction. However, emerging data raises concerns about a potential increase in AF risk associated with its use. Real-world data report notably higher incidence of newly diagnosed AF in patients treated with mavacamten (11% over median follow up of 8 months, with a median time to event of 26 weeks) compared to phase 3 trials of mavacamten in obstructive HCM (EXPLORER-HCM and VALOR-HCM), where rates were 2% over 30 weeks and 3.6% over 56 weeks, respectively [103–105]. The real-world cohort, however, included a higher prevalence of pre-existing AF, suggesting potential selection bias towards a higher risk population.

Mavacamten has also been linked to a reduction in LA systolic function in some studies. Its negative inotropic effects on the atrial myocardium may contribute to AF susceptibility, as LA volumes remain unchanged despite reductions in mitral regurgitant volume [106], potentially offsetting the benefits observed with improved LV filling pressures and reductions in NT-proBNP. Conversely, the VALOR-HCM study demonstrated significant improvement in LA strain and volume, pointing towards beneficial effect of mavacamten on LA function [107]. Although computational modelling suggests that mavacamten improves certain electrophysiological parameters, outperforming metoprolol in alleviating calcium overload [108], these benefits do not extend to reducing the risk of AF in patients. These findings underscore the need for caution and close monitoring when prescribing mavacamten in patients with a higher predisposition to AF.

New benefits of existing treatments have also been identified. For instance, diltiazem, often used as a rate controlling agent, showed benefit in a randomised clinical trial in preclinical HCM in MYBPC3 mutation carriers by attenuating early LV remodelling. In Troponin T mutation mice, it prevented isoproterenol-induced acute heart failure without affecting heart rate, likely via modulating calcium influx [109]. Similarly ranolazine, a late sodium current inhibitor, mitigates ion channel abnormalities seen in HCM by reduction of calcium overload and stabilisation of ryanodine receptors [110]. It has been shown to not only improve anginal symptoms but also reduce ventricular arrhythmic burden and was well-tolerated and safe, though safety data in HCM remains limited [111].

### Diagnosis and prediction of atrial arrhythmias

AF in HCM is frequently subclinical, with device-detected AF occurring at a rate of 7% per year - tenfold higher than symptomatic AF [112]. The HCM-AF score facilitates risk stratification by incorporating LA size, age at evaluation, age at diagnosis, and presence of heart failure [113]. Those at high-risk require more intensive surveillance, such as through implantable loop recorders, which have been shown to increase AF detection rates over threefold. However the retrospective nature of the analysis necessitates a cautious interpretation [114].

Imaging may support the identification of patients at risk of AF. For example, LA dilatation, reflecting chronic pressure overload and diastolic dysfunction, correlates with the risk of AF in patients with HCM [115]. Further, even in patients with normal LA size, reduced LA strain correlates with increased risk of AF, suggesting it may be a marker of subclinical atrial dysfunction [116]. Nevertheless, imaging parameters alone may not be sufficient to fully capture the arrhythmogenic substrate, prompting interest in functional and electrocardiographic predictors of arrhythmia risk.

One such marker is P wave dispersion – the difference between the maximum and minimum P wave duration on ECG – which reflects atrial conduction heterogeneity and has been associated with increased risk of AF [117]. Similarly, P wave duration ≥ 140ms, particularly when combined with LA dilatation, is also predictive of AF [118].

Integrating imaging and ECG data may offer a more nuanced insight into atrial remodelling. The PA-TDI interval, measured from the onset of the P wave on ECG to the peak lateral A’ velocity on tissue Doppler, represents the time from electrical activation to the onset of mechanical contraction, effectively quantifying atrial conduction time. Prolongation of this interval, caused by atrial enlargement or conduction delay, has been associated with higher AF incidence even in the absence of overt structural changes [119].

## Conclusion

Atrial remodelling in HCM, driven by complex molecular, cellular, and structural changes, is a critical determinant of the arrhythmogenic potential observed in this condition. The interplay between hypertrophy, fibrosis, and altered electrophysiological properties in the left atrium leads to a progressive substrate for AF, which significantly impacts patient outcomes. Recent advances in electrophysiology, high-resolution imaging, and molecular biology have provided unprecedented insights into the pathophysiology of HCM, particularly the mechanisms driving atrial remodelling and arrhythmogenesis. These developments enhance our understanding of disease progression and offer opportunities for earlier diagnosis and more targeted interventions. As we move toward a precision medicine approach, integrating novel diagnostic tools and therapies holds promise for transforming outcomes in this challenging population.

## Data Availability

No datasets were generated or analysed during the current study.
